# Predictive factors for time to recurrence, treatment and post-recurrence survival in patients with initially resected colorectal liver metastases

**DOI:** 10.1186/s12957-015-0738-8

**Published:** 2015-12-03

**Authors:** Jon-Helge Angelsen, Asgaut Viste, Inger Marie Løes, Geir Egil Eide, Dag Hoem, Halfdan Sorbye, Arild Horn

**Affiliations:** Department of Acute and Digestive Surgery, Haukeland University Hospital, N-5021 Bergen, Norway; Department of Clinical Medicine, University of Bergen, Bergen, Norway; Department of Oncology, Haukeland University Hospital, Bergen, Norway; Centre for Clinical Research, Haukeland University Hospital, Bergen, Norway; Department of Global Public Health and Primary Care, University of Bergen, Bergen, Norway; Department of Clinical Science, University of Bergen, Bergen, Norway

**Keywords:** Resection colorectal liver metastases, Overall survival, Time to recurrence, Sites of recurrence, Perioperative chemotherapy, Post-recurrence survival

## Abstract

**Background:**

Despite progress in resection for colorectal liver metastases (CLM), the majority of patients experience recurrence. We aimed to evaluate factors influencing time to recurrence (TTR), treatment and post-recurrence survival (PRS) related to site of recurrence.

**Methods:**

This is a retrospective population-based cohort study (1998–2012) of consecutive patients without extrahepatic disease treated with resection for CLM in a referral centre.

**Results:**

A total of 311 patients underwent resection for CLM. After a median follow-up of 4.2 years (range 1.2–15.2), 209 (67.4 %) patients developed recurrence, hepatic 90, extrahepatic 59 and both 60. Median TTR was 14.0 months, and 5-year recurrence-free status was 25.7 %. Five- and 10-year overall survival (OS) was 38.8 and 22.0 %, respectively. Median OS was 45 months. A multivariate analysis displayed synchronous disease (hazard ratio (HR) 1.50), American Society of Anaesthesiologists (ASA) score (HR 1.40), increasing number (HR 1.24) and size of metastases (HR 1.08) to shorten TTR (all *p* < 0.05). Perioperative chemotherapy (*n* = 59) increased overall TTR (HR 0.63) and overall survival (OS; HR 0.55). Hepatic TTR was correlated to synchronous disease (HR 2.07), number of lesions (HR 1.20), R1 resection (HR 2.00) and ASA score (HR 1.69), whereas extrahepatic TTR was correlated to N stage of the primary (HR 1.79), number (HR 1.27) and size of metastases (HR 1.16). Single-site recurrence was most common (135 of 209, 64.5 %), while 58 patients had double- and 16 triple-site relapses. Median PRS was 24.3 months. There was a difference in median PRS (months) according to site of relapse: liver 30.5, lung 32.3, abdominal 22.0, liver and lung 14.3, others 14.8 (*p* = 0.002). Repeated liver resections were performed in *n* = 57 patients resulting in 40.6 months median OS and 36.8 % 5-year OS.

**Conclusions:**

An adverse overall TTR was correlated to number and size of metastases, ASA score and synchronous disease. Perioperative chemotherapy increased TTR and OS after surgery for CLM. Patients with solitary post-resection relapse in the liver or lungs had the potential for longevity due to multimodal treatment.

## Background

Surgical intervention (resection or local ablation) is the only potentially curative option for patients with colorectal liver metastases (CLM). Due to progress in surgical technique and perioperative care during the last two decades, perioperative morbidity (17–38 %) and mortality (1–2 %) have declined [1–3]. Furthermore, by multimodal treatment like chemotherapy, radiofrequency ablation (RFA) and portal vein embolization, some patients have achieved downsizing of initially unresectable CLM and might be offered a potentially curative resection [4]. Perioperative chemotherapy has also improved progression-free survival [5] and overall survival (OS) in the adjuvant setting [6]. Following these advancements, OS has increased to 47–58 % in several series [3, 7, 8]. Despite this, the recurrence rates (57–77 %) and disease-free survival have remained almost unchanged in the same period [7, 9–11]. Due to improved surgical approach, repeated resections are more often offered to selected patients with recurrent disease [12, 13]. For patients beyond the range of cure, optimal oncological therapy may yield life extension [14, 15].

A large number of reports have evaluated survival after resection, whereas rather few studies have highlighted the fate of patients with recurrence according to site of relapse.

In this paper, we aimed to analyse the (1) sites of recurrence after liver resections for CLM, (2) factors influencing time to recurrence (TTR) in different sites and (3) treatment of post-resection recurrence and the impact on survival according to site of relapse.

## Methods

This is a population-based retrospective cohort study with a consecutive series of patients with CLM treated at Haukeland University Hospital, Norway (1998–2012). The data were retrospectively recorded from 1998 to 2008 and prospectively from 2009 to 2012. The unit is the only hepato-pancreato-biliary centre in the region, which makes this a population-based cohort from this catchment area of 0.7 million people. Clinical data were retrieved from the patients’ medical records. All patients were prospectively followed up until 15 March 2014. Recorded variables were TNM stage and site of primary tumour, synchronous metastases (detected within 3 months after resection of the primary colorectal tumour [16, 17]), time between resection of primary tumour and diagnosis of liver metastases (disease-free interval), number and size of metastases, chemotherapy (indication, number of cycles and response), American Society of Anaesthesiologists (ASA) score, date of liver resection, resection margins (R1 <1 mm [8]), complications (Clavien-Dindo classification [18]), in-hospital mortality, time to recurrence, sites of recurrence and death (perioperative, cancer-related and other causes) and last date of follow-up for survivors.

### Preoperative assessments

**Fig. 1:**
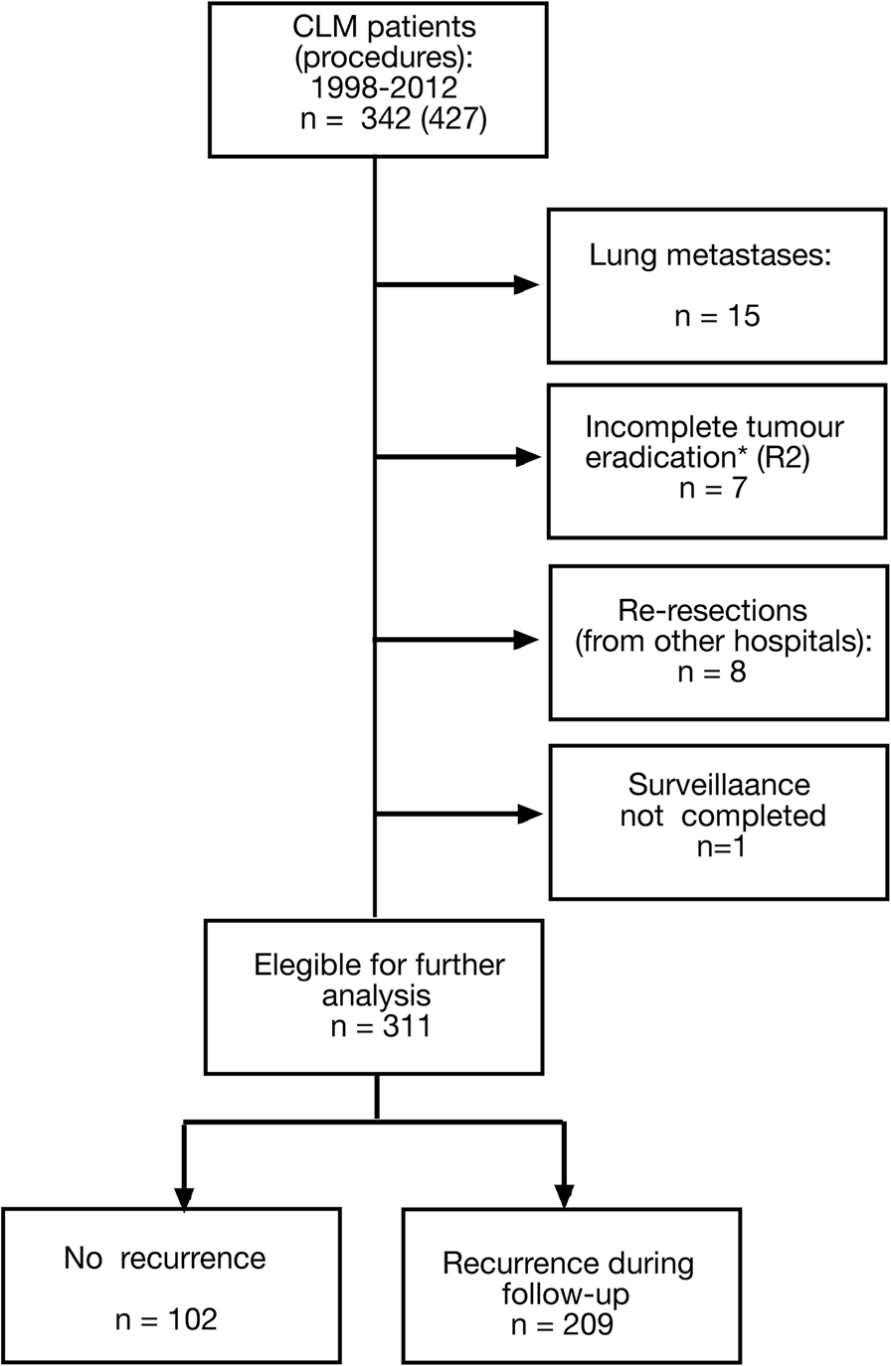
Selection of patients. Incomplete tumour eradication: liver, *n* = 3 (i.e. failure of completing two-stage resection); primary rectal tumour not resected due to progression after liver first procedure, *n* = 3; minor liver resection performed despite finding of peritoneal carcinomatosis, *n* = 1

The selection criteria for surgery were a sufficient tumour-free liver remnant (>30 %) and absence of (a) disseminated disease as evaluated preoperatively and/or (b) non-resectable extrahepatic metastases. Patients with preoperatively detected resectable extrahepatic disease and macroscopically incomplete resection (R2) were excluded from further analyses (Fig. 1). Preoperative investigations included computed tomography (CT) scan of the chest and abdomen/pelvis and tumour marker analysis (carcinoembryonic antigen (CEA)). In cases with an inconclusive CT scan, magnetic resonance imaging (MRI) of the liver, contrast-enhanced ultrasound and/or ^18^fluorodeoxyglucose (FDG) positron emission tomography (PET)/CT scan were performed. Each patient was finally discussed in a multidisciplinary team setting.

### Chemotherapy

Perioperative chemotherapy was given in 59 cases. Forty-six patients were initially considered unresectable and underwent downsizing chemotherapy. Seven patients developed CLM while on adjuvant chemotherapy after resection for stage III colon cancer. The Response Evaluation Criteria in Solid Tumour (RECIST) version 1.1 was applied to evaluate the efficiency of chemotherapy [19]. The size of the metastases was measured on CT scan by dedicated radiologists. All patients in the perioperative group received treatment with FOLFOX regimen (fluorouracil, leucovorin and oxaliplatin) with intended six cycles before and after surgery. The indications for perioperative chemotherapy have changed during the study period. A total of 17 patients were enrolled in the European Organisation for Research and Treatment of Cancer (EORTC) multicentre study 40983 in the period 2001–2004 [5]. Later on, perioperative chemotherapy given as Nordic FLOX [20] were offered to patients <76 years with Eastern Cooperative Oncology Group (ECOG) performance status ≤1 and no previous treatment with oxaliplatin. In the downsizing group, patients were treated with a variety of chemotherapy regimens. First-line treatment with Nordic FLOX or Nordic FLIRI regimen was most commonly used [15, 20] optionally in combination with endothelial growth factor receptor (EGFR) inhibitors (if *KRAS* wild type) or angiogenesis inhibitors.

### Surgical procedures

Surgical techniques included intraoperative ultrasonography, repeated inflow control (Pringle manoeuvre) and transection using Ultracision, Kelly clamp, Cavitron Ultrasonic Surgical Aspirator (CUSA) or Ultrasonic Aspirator (Olympus Sonosurg™). Throughout the period, we have intended to obtain a parenchyma sparing approach with wedge resections whenever possible. Formal resections (hemihepatectomies or lobectomies) were reserved for metastases abutting the portal triad or the hepatic veins. To increase intended complete tumour eradication, intraoperative RFA (StarBurst**®**) and portal vein ligations/embolization with two-stage resections were performed. Simultaneous colorectal cancer surgery was reserved for healthy patients with colon cancer and less advanced CLM. Further details are listed in Table 1.

**Table 1:** Clinical characteristics

Variable, statistics	Estimate
Age in years, median (range)	66.1 (22.8, 91.3)
Gender males/females—ratio	169/142
Location primary tumour	
Colon, *n* (%)	205 (65.9)
Rectum, *n* (%)	101 (32.5)
Combined, *n* (%)	5 (1.6)
Synchronous metastases^a^, *n* (%)	157 (50.5)
Disease-free interval^b^ in months, median (range)	4.2 (−11, 131)
Resections, *n*	311
Hemihepatectomy/lobectomy, *n* (%)	137 (44.1)
Wedge/segment resections, *n* (%)	174 (55.9)
Simultaneous radio frequency ablation, *n* (%)	12 (3.9)
Two-stage resections with PVL, *n* (%)	3 (1.0)
Simultaneous colorectal cancer surgery, *n* (%)	18 (5.8)
R1 resections (<1 mm)	63 (20.3)
Extent of RM in millimeter, median (range)	4 (0–50)
Number of metastases, median (range)	2 (1, 12)
Metastases diameter in centimeter, median (range)	3.0 (0.2, 15.0)
Bilobar metastases, *n* (%)	108 (34.7)
Number of procedures/patient (1/2/3/4/5)	254/42/11/3/1
Morbidity (Clavien-Dindo score 1/2/3/4/5)	19/28/52/5/6
Outcome of chemotherapy (RECIST criteria)	
Partial response, *n* (%)	63 (56.2)
Stable disease, *n* (%)	42 (37.5)
Progression, *n* (%)	5 (4.4)
Unknown, *n* (%)	2 (1.8)

Unknown Clavien-Dindo score *n* = 1

*RM* resection margin, *RECIST* Response Evaluation Criteria in Solid Tumour version 1.1

^a^Synchronous metastases: detection of liver metastases within 3 months after primary colorectal resection

^b^Disease-free interval: time between resection of the primary colorectal tumour and detection of CLM

### Surveillance

Follow-up after surgery included CT scan of the chest, abdomen and pelvis every 3 months for the first 2 years, and thereafter every 6 months for the next 3 years. After completing the 5-year follow-up, survival data were retrieved from the medical record and the Norwegian National Registry. Patients that died from other causes were also included in the analysis of OS but were censored in the estimation of TTR according to the definition stated by Punt et al. [21].

### Statistical analysis

Variables with possible impact on TTR and OS like size and number of metastases, resection margins, synchronous disease and TNM stage of primary tumour were analysed with univariate and multivariate survival methods [22]. The exact chi-square (*χ*^2^) test was used for categorical variables, the *t* test and the one-way analysis of variance for normally distributed variables, and the Mann-Whitney *U* test and the Kruskal-Wallis test for non-normally distributed continuous variables. Univariate analyses of TTR and OS were estimated by the Kaplan-Meier method [23] and tested for significance with the log-rank test [24]. Multivariate analyses of risks for overall, hepatic and extrahepatic TTR were performed as Cox proportional hazards regression reporting hazard ratios (HR) and 95 % confidence intervals (CI) [25] A *p* value ≤0.05 was considered significant. OS was defined as time to death irrespective of cause, and TTR was defined as the interval between resection and the detection of relapse [21]. The analyses were performed using SPSS Statistics version 22 (IBM Corp., Armonk, NY, USA) and Stata 13 statistical software (StataCorp, College Station, TX, USA). We decided to use TTR rather than disease-free survival as an outcome in assessing recurrence patterns, since the latter has treatment-related and non-cancer-related deaths as endpoints [21].

### Ethics

The regional committee for medical and health research ethics, western Norway approved the study, with an exemption to the requirement for obtaining informed consent from patients included in the retrospective part (1998 to 2008). In the prospective part (2009 to 2012), patients were enrolled through written consent.

## Results

A total of 342 patients were resected for CLM of whom 311 were eligible for further analysis. Patient selection and characteristics are outlined in Fig. 1 and Table 1, respectively.

### Patterns and sites of recurrence

**Fig. 2:**
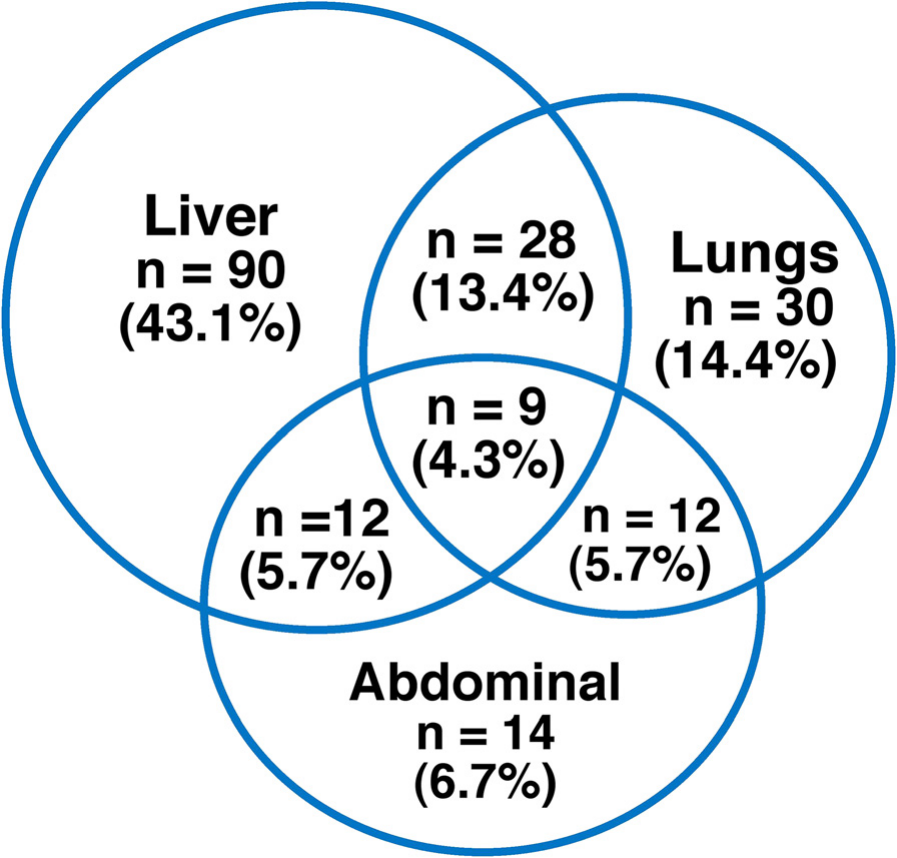
Patterns of recurrence in the three most common sites in *n* = 209 patients. Note: Other sites and combinations of recurrence: cerebral only, *n* = 1; liver/bones, *n* = 2; liver/cerebral, *n* = 1; lungs/cerebral, *n* = 2; liver/lungs/cerebral, *n* = 1; liver/abdominal/bones, *n* = 2; liver/lungs/bones, *n* = 4; liver/ovary *n* = 1. Recurrence was not detected in *n* = 101 patients with a median observation time of 4.2 years. Definitions: *Abdominal* locoregional recurrence involving peritoneal, lymph node and local recurrence

After a median follow-up of 4.2 years (range 1.2–15.2) 209 patients (67.4 %) developed recurrence. The sites of recurrence were distributed between hepatic (*n* = 90), extrahepatic (*n* = 59) and both locations (*n* = 60). Further details are outlined in Fig. 2. Median TTR was 14.0 months, and 5-year recurrence-free status was 25.7 %. Single-site relapse was most common (135 of 209, 64.5 %), while 58 patients had double- and 16 triple-site relapses. TTR was associated with number and size of metastases, synchronous disease, increasing ASA score and perioperative chemotherapy in the multivariate analysis (Tables 2 and 3). Hepatic TTR correlated with synchronous CLM, ASA score, R1 resections and number of metastases. Extrahepatic TTR (including hepatic/extrahepatic) corresponded with node positive of the primary, number and size of metastases. Positive margins and synchronous disease were insignificant. The sites of recurrence were independent on the primary tumour location (colon vs. rectum) both in univariate and multivariate analyses.

**Table 2:** Cox regression analysis of factors affecting time to overall recurrence

		Overall recurrence (TTR)					
		Univariate			Multivariate		
Variable	*n*	HR	95 % CI	*p*	HR	95 % CI	*p*
Age/10 years	311	1.11	(0.98, 1.26)	0.113	1.08	(0.94, 1.25)	0.253
Synch. mets							
No	154	1.00	Reference	0.002	1.00	Reference	0.010
Yes	157	1.65	(1.25, 2.17)		1.50	(1.10, 2.04)	
No. of mets	311	1.21	(1.14, 1.30)	<0.001	1.24	(1.14, 1.34)	<0.001
Mets diam (cm)	311	1.11	(1.04, 1.18)	0.002	1.08	(1.01, 1.16)	0.022
RM status							
R0	246	1.00	Reference	0.013	1.00	Reference	0.143
R1	63	1.53	(1.11, 2.11)		1.30	(0.92, 1.84)	
ASA score (1–3)	311	1.28	(1.00, 1.65)	0.054	1.40	(1.05, 1.87)	0.022
Clavien-Dindo	311	1.12	(1.01, 1.25)	0.037	1.07	(0.95, 1.20)	0.267
N-pos^a^							
No	190	1.00	Reference	0.058	1.00	Reference	0.091
Yes	109	1.32	(0.99, 1.78)		1.30	(0.96, 1.77)	
Chemotherapy							
None	199	1.00	Reference	0.010	1.00	Reference	0.014
Periop.	59	0.71	(0.49, 1.05)		0.63	(0.42, 0.95)	
Downsiz.	46	1.31	(0.90, 1.89)		0.93	(0.62, 1.39)	
Adj. colon^b^	7	2.98	(1.31, 6.79)		2.80	(1.21, 6.48)	

Unknown N status *n* = 12. Unknown RM *n* = 2

*RM* resection margins, *HR* hazard ratio, *CI* confidence interval

^a^N-pos: positive lymph nodes of the primary tumour in colon or rectum

^b^Adj. colon: adjuvant chemotherapy after surgery for stage III (node positive) colon cancer

**Table 3:** Cox regression analysis of factors affecting time to hepatic and extrahepatic recurrence

	Hepatic recurrence			Extrahepatic recurrence^a^		
	Multivariate					
Variable	HR	95 % CI	*p*	HR	95 % CI	*p*
Age/10 years	1.00	(0.81, 1.24)	0.995	1.14	(0.95, 1.37)	0.165
Synch. mets						
No	1.00	Reference	0.002	1.00	Reference	0.499
Yes	2.07	(1.28, 3.33)		1.15	(0.77, 1.73)	
No. of mets	1.20	(1.07, 1.35)	0.005	1.27	(1.14, 1.41)	<0.001
Mets diam (cm)	0.99	(0.88, 1.10)	0.828	1.16	(1.07, 1.27)	0.001
RM status						
R0	1.00	Reference	0.010	1.00	Reference	0.769
R1	2.00	(1.20, 3.31)		0.93	(0.57, 1.52)	
ASA score						
(1–3)	1.69	(1.08, 2.64)	0.021	1.24	(0.85, 1.81)	0.264
Clavien-Dindo	0.99	(0.83, 1.19)	0.958	1.12	(0.96, 1.30)	0.165
N-pos						
No	1.00	Reference	0.652	1.00	Reference	0.007
Yes	0.90	(0.58, 1.41)		1.79	(1.16, 2.76)	
Chemotherapy						
None	1.00	Reference	0.143	1.00	Reference	0.036
Periop.	0.63	(0.34, 1.18)		0.64	(0.37, 1.11)	
Downsiz.	1.36	(0.78, 2.39)		0.63	(0.35, 1.14)	
Adj. colon	2.28	(0.54, 9.60)		3.12	(1.10, 8.84)	

Unknown N status *n* = 12. Unknown RM *n* = 2

*RM* resection margins, *HR* hazard ratio, *CI* confidence interval

^a^Extrahepatic recurrence also included combined hepatic and extrahepatic recurrence

### Chemotherapy

**Fig. 3:**
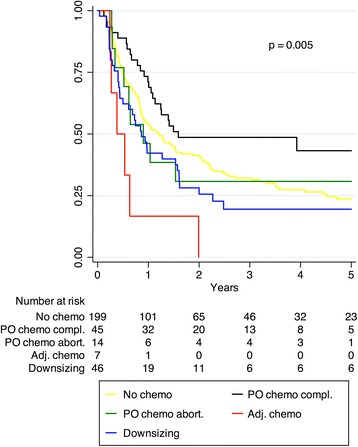
Time to recurrence according to different chemotherapy regimens. *PO chemo compl* perioperative chemotherapy completed, *Adj. chemo* adjuvant chemotherapy after stage III colon cancer (lymph node positive) with progression of liver metastases. Log-rank test: No chemo vs. PO chemo completed, *p* = 0.045; No chemo vs. downsizing chemo, *p* = 0.155

The clinical characteristics of patients undergoing downsizing or perioperative chemotherapy vs. surgery alone are described in Table 4. Forty-five patients completing perioperative chemotherapy experienced a longer median TTR (19.1 months) compared with patients who aborted this treatment (*n* = 14, 10.8 months), downsizing chemotherapy (*n* = 46, 10.4 months), surgery alone (*n* = 199, 14.4 months) or adjuvant chemotherapy after resection of the primary (*n* = 7, 4.6 months), *p* = 0.005. Five-year recurrence-free status for these groups was 43.2, 30.8, 19.5, 23.7 % and none, respectively, (*p* = 0.005, Fig. 3). TTR was different between patients with response, stable disease and progression, with a 5-year recurrence-free status of 35.3, 26.0 % and none, respectively (*p* = 0.021). Positive margins influenced TTR in responders to chemotherapy where 3-year recurrence-free status in R0 and R1 was 47.8 and 7.1 % and median TTR was 2.0 and 0.4 years, respectively (*p* < 0001). This difference was not evident in patients with stable disease. For this group, 3-year recurrence-free status was 30.4 % in R0 whereas all patients with R1 had recurrence within 3 years. Median TTR was 1.1 and 1.5 years, respectively (*p* = 0.756). Perioperative chemotherapy correlated to an increased overall TTR in the multivariate analysis (Tables 2 and 3). Five- and 10-year OS with perioperative chemotherapy vs. surgery alone was 57.0 and 31.6 % vs. 37.1 and 20.0 %, respectively (*p* = 0.024). Median OS in the same groups was 73 and 43 months, respectively. This finding was also confirmed in a multivariate analysis (HR 0.55 [0.34, 0.89], *p* = 0.014). Patients completing perioperative regimen had an improved 5- and 10-year OS of 62.0 and 51.6 %, respectively.

**Table 4:** Clinical characteristics in *n* = 304 patients with no chemotherapy, perioperative and downsizing chemotherapy in conjunction with resection for colorectal liver metastases

Variable	No chemo (*n* = 199)	Perioperative (*n* = 59)	Downsizing (*n* = 46)	*p*
No. of mets (mean/median)	2.1/1	2.4/2	3.4/3	0.001^a^
Mets size (cm, median)	3.5/3	2.8/2.1	4.1/3.5	0.003^a^
Age (year, mean/median)	65.9/66.5	63.7/65.1	62.0/63.6	0.055^b^
R1 (*n*, %)	40 (20.2)	12 (20.7)	10 (21.7)	0.973^c^
N-pos primary (*n*, %)	68 (36.0)	19 (32.8)	21 (46.7)	0.311^c^
Clavien-Dindo (1–5)	61 (31.3)	24 (41.4)	16 (35.6)	0.350^c^
ASA (1/2/3)	20/136/43	5/50/4	2/34/10	0.069^c^
Synch. mets (*n*, %)	71 (35.7)	36 (61.0)	30 (65.2)	0.001^c^
Gender (M/F)	104/95	36/23	26/20	0.475^c^
Recurrence pattern (*n*, A/B)	53/82	15/18	20/15	0.256^c^

Seven of 311 patients had progress on chemotherapy after stage III colon cancer, not included in the analysis

*A* hepatic recurrence, *B* extrahepatic recurrence

^a^Kruskal-Wallis test

^b^One-way analysis of variance

^c^ *χ* ^2^ test

### Post-recurrence survival (PRS)

**Fig. 4:**
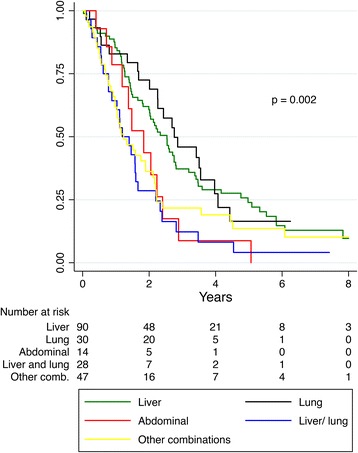
Post-recurrence survival according to sites of relapse. Log-rank test: recurrence in lung vs. liver, *p* = 0.586; liver vs. abdominal, *p* = 0.040; liver vs. liver/lung, *p* = 0.010; liver vs. other combinations, *p* = 0.012

Median PRS was 24.3 months and differed according to sites of relapse; liver 30.4; lungs 33.1; abdominal 22.0; liver and lungs 14.3; other combinations 14.8 months as outlined in Fig. 4 (*p* = 0.002). Five-year PRS in these groups was 23.9, 16.4, 8.7, 4.1 and 13.6 %, respectively. Median PRS was related to the number of recurrence sites; one site 28.8; two 16.8; three 13.5 months (*p* = 0.001). Hepatic re-resections were performed in *n* = 57 cases, whereas 9 patients had resections of emerging lung metastases. The number of recurrence sites correlated with a secondary surgical resection (*p* < 0.001). Of 90 patients with sole hepatic recurrence, 48 underwent resection, 20 chemotherapy, 6 RFA and 9 patients best supportive care. Data were not available in 7 patients. Median PRS (months) varied between these groups; resection 50.0; chemotherapy 15.2; RFA 19.9; best supportive care 5.3 (*p* < 0.001). Patients with combined recurrence in the liver and lungs underwent resection in 5 of 28 (17.8 %) cases.

### Overall survival

Three-, 5- and 10-year OS after the first hepatic resection were 58.8, 38.8 and 22.0 %, respectively. Median OS was 45 months. During follow-up, 17 non-CLM-related deaths were observed (other malignant disease *n* = 2; cardiac disease *n* = 4; other liver disease *n* = 2; miscellaneous *n* = 9). Five-year OS after the second liver resection was 36.8 % and median OS 40.6 months.

## Discussion

Single-site hepatic and pulmonic recurrences were most common after surgery for CLM. Positive resection margins, number of metastases and synchronous disease were associated with hepatic recurrence, whereas number and size of metastases and positive primary nodal status were correlated with extrahepatic recurrence. Perioperative chemotherapy increased TTR and OS as well. Patients with single-organ recurrence in the lungs or liver were offered re-resections and/or supplementary chemotherapy had an extended survival.

About two thirds experienced recurrence after hepatic resection with the liver as the most common site (43 %) which is fairly consistent with other reports [7, 9, 10]. Positive margins were associated with hepatic TTR, supporting previous series from de Jong et al. where R1 resection was predictive for hepatic recurrence (HR 1.36) [7]. In a former publication, we also demonstrated a correlation between R1 resections and local recurrence [26]. In cases with synchronous disease, sole hepatic post-resection recurrence may indicate an underestimation of tumour advancement due to preoperatively undetected liver lesions. A limitation in our cohort is the lack of routinely performed preoperative MRI. Several series have shown this modality to be more accurate than CT scan [27–30]. With the evolvement of efficient cytotoxic regimens, MRI may also yield additional information in cases of CT-verified complete response [30]. Primary nodal status indicated a high risk for extrahepatic recurrence. This represents most probably a more aggressive clinical course with undetected systemic disease at the time of liver surgery. A recent study by Lee et al. detected a significant distributive variation in metastatic pattern, concatenating a rectal primary with extrahepatic recurrence, and a colon primary with hepatic recurrence, respectively [31]. No such association was verified in the present study.

The indication for perioperative chemotherapy is still reported as controversial [32]. The EORTC intergroup trial 40983 demonstrated an increase in progression-free survival in patients undergoing perioperative chemotherapy [5], especially in patients with CEA above 5.0 [33]. However, after a long-term follow-up, no significant benefit was obtained in OS [34]. The current study observed a significant improvement in overall TTR for the perioperative chemotherapy group compared with resection only. This finding was not significant in the site-specific recurrence analysis (Tables 2 and 3), most probably due to an insufficient number of patients. Data from the EORTC trial also demonstrated a reduction in hepatic relapse following perioperative FOLFOX regimen [35]. In our cohort, patients in the perioperative group had more advanced clinical course like synchronous disease and increased number of metastases compared to patients with resection only. Positive margins influenced TTR in cases with response (*p* < 0.0001) as opposed to patients with stable disease (*p* = 0.756). In responders after chemotherapy, tumour shrinkage may lead to remaining therapy-resistant islets of malignant cell clusters near the main lesion as well as an irregular surface which may cause local recurrence in cases with narrow or positive margins [36]. Furthermore, we detected a significantly better 5-year OS in the chemotherapy group, with a 5-year OS of 62 % in patients completing chemotherapy, as opposed to other reports [37, 38]. However, our results should be interpreted with caution due to possible selection bias and a heterogeneous cohort mainly retrospectively observed.

Patients in the downsizing group presented an insignificant difference in the univariate (*p* = 0.155) and multivariate analysis in overall, hepatic and extrahepatic TTR compared with the surgery alone group (Fig. 3 and Tables 2 and 3) despite more adverse tumour load (Table 4). These results support a previous study by Adam et al. [4]. Patients offered resection after progression while on adjuvant chemotherapy after stage III colon surgery demonstrated a short median TTR of 4.6 months. Based on this finding in this small group of patients, surgery may not be beneficial [39].

We demonstrated that PRS was correlated to the site of recurrence. Single-organ lesions in the lungs or liver appeared to have the best outcome. A high proportion of repeated hepatic resections increased the PRS in patients with hepatic recurrence. Five-year OS of 36.8 % after the second resection was comparable to the survival rates after the first resection (5-year OS 38.8 %). A similar survival rate has been demonstrated in several other studies [12, 13, 40]. The use of repeated liver resections varies in the literature. Assumpcao et al. [11] and D’Angelica et al. [9] performed a second resection in 28 and 30 % of the cases with recurrence, respectively, whereas Mise et al. [10] conducted metastasectomy in 85 % of isolated hepatic or lung recurrence. Despite unresectable lesions in the lung, nearly 3-year median survival was observed in the present cohort. This finding may also justify the expanding criteria for liver resection in selected patients with unresectable lung metastases [41]

## Conclusions

Sites of recurrence predict the outcome after surgery for CLM. Resection margins, number of metastases and synchronous disease were associated with hepatic recurrence, whereas N positive (primary tumour), number and size of metastases were associated with extrahepatic recurrence. Perioperative chemotherapy prolonged TTR and increased OS significantly. Patients with single-organ relapse have the potential for longevity due to multimodal treatment with repeated resections and supplementary chemotherapy.

## Abbreviations

ASA: American Society of Anaesthesiologists
CLM: colorectal liver metastases
HR: hazard ratio
OS: overall survival
PRS: post-recurrence survival
RFA: radio frequency ablation
TTR: time to recurrence
